# The P2X7 receptor: insights from the murine monoclonal antibody clone L4

**DOI:** 10.1007/s11302-026-10176-0

**Published:** 2026-07-24

**Authors:** Ronald Sluyter, Amal Elhage, Rachael Bartlett, Debbie Watson, Leanne Stokes, Kristen K. Skarratt, Stephen J. Fuller, Ben J. Gu

**Affiliations:** 1 Molecular Horizons and School of Science, University of Wollongong, Wollongong, NSW Australia; 2 School of Chemistry, Pharmacy & Pharmacology, University of East Anglia, Norwich, Norfolk, UK; 3 Sydney Medical School Nepean, Faculty of Medicine and Health, The University of Sydney, Nepean Hospital, Penrith, NSW Australia; 4 Qankorey Biotechnology Co. Ltd, Changsha, China; 5 The Florey Institute, University of Melbourne, Parkville, VIC Australia; 6 The Innate Phagocytosis Lab, Melbourne, VIC Australia

**Keywords:** Bone cell, Cancer cell, Leukocyte, Neural cell, *P2RX7*, Skin cell

## Abstract

The P2X7 receptor (or P2X7) is an ATP-gated ion channel with important roles in the immune and other body systems. Much has been garnered about P2X7 using the first published anti-human P2X7 monoclonal antibody (mAb) (clone L4). This mAb can be used to impair P2X7 channel and pore activity and events downstream of P2X7 activation in vitro and in vivo. Immunolabelling with this mAb has been used in flow and mass cytometry, confocal microscopy and immunohistochemistry to help establish the presence of P2X7 on human leukocytes and other primary cell types, as well as various malignant cell lines. This mAb has been applied to immunoprecipitate P2X7 to identify co-associated molecules to help uncover novel roles of the receptor such as phagocytosis. Recently, this mAb has been shown to mediate complement-dependent cytotoxicity, offering a new avenue of limiting P2X7-mediated processes by depleting P2X7^+^ cells. Finally, this mAb has been used to identify and characterise polymorphic and other variants of P2X7. This article will discuss the origins and various applications of the anti-P2X7 mAb (clone L4). As an early user of this mAb and pioneer of other purinergic technologies, this article is a tribute to the late Francesco Di Virgilio.

## Introduction

The P2X7 receptor (or P2X7) is an ATP-gated ion channel belonging to the family of P2X receptors [1]. As illustrated in highly cited reviews by the late Francesco Di Virgilio [2] and his colleagues, P2X7 plays important roles in inflammation [3] and immunity [4], including the stimulation of the NOD-like receptor protein 3 (or NLRP3) inflammasome [5] and subsequent processing and release of interleukin (IL)−1 family cytokines [6]. As also reviewed by Di Virgilio and colleagues, P2X7 can play opposing roles in cell proliferation and death [7] and in cancer resistance and progression [8].

As a pioneer of new technologies to study P2X7 and other facets of purinergic signalling, perhaps most notably the development of luciferase-based systems to measure extracellular ATP [9], Di Virgilio was an early adopter of the first published anti-human P2X7 monoclonal antibody (mAb) (clone L4) [10], hereafter termed the L4 mAb. In these early studies, Di Virgilio and colleagues used this mAb to establish the role of P2X7 in the fusion of macrophages to form giant cells [11, 12], and to establish the presence of P2X7 on human dendritic cells (DCs) including its role in ATP-induced cytokine release [13]. As part of a special issue in tribute to the late Francesco Di Virgilio [2], this article will describe the origins of the L4 mAb and how this mAb has been used to investigate P2X7. This includes how the L4 mAb has been applied to delineate the presence and functions of P2X7 in different cell types, largely immune cells, and to establish various roles of this receptor in health and disease, most commonly within the immune system.

### Origins of the anti-human P2X7 mAb (clone L4)

The L4 mAb was developed by Gary Buell, Iain Chessell and colleagues at the end of the twentieth century [10]. This mAb was established by repeated immunisation of Balb/c mice with human P2X7-transfected Balb/c myeloma XS63 cells and standard hybridoma techniques. This culminated in a murine IgG_2b_ mAb that bound to human P2X7 but not to human P2X1 or P2X4 expressed in HEK293 cells as assessed by flow cytometry. The screening of this mAb against human P2X1 and P2X4 was somewhat serendipitous, with these two P2X subtypes along with P2X7 being the best characterised P2X subtypes within immune cells [14, 15]. These expression and activity studies in human P2X7-transfected HEK293 cells (HEK-hP2X7 cells) also indicated that the L4 mAb binds to the extracellular domain of P2X7 [10]. In this original study, the L4 mAb was also observed to bind P2X7 in cultured human peripheral blood-derived monocytes by flow cytometry and cells of dendritic morphology in human tonsils by immunohistochemistry. Moreover, this mAb impaired 2'(3')-*O*-(4-benzoylbenzoyl)ATP (BzATP)-induced inward currents in HEK-hP2X7 cells but not in HEK293 cells transfected with murine or rat P2X7 or human P2X3 or P2X4. This mAb also impaired BzATP-induced IL-1β release from lipopolysaccharide (LPS)-primed human monocyte THP-1 cells. Finally, this mAb was shown to immunoprecipitate human P2X7 from biotinylated XS63 cells transfected with human P2X7 but was unable to detect denatured human P2X7 in cell lysates by immunoblotting. Detailed protocols for some applications with the L4 mAb have been published by others [16–19]. The L4 mAb (deposited by Ben Gu, RRID:AB_2618074) is commercially available as a hybridoma supernatant or as growing or frozen cells from the Developmental Studies Hybridoma Bank (or DSHB) (Iowa City, USA).

### Functional studies with the anti-human P2X7 mAb (clone L4)

#### Channel and pore activity and downstream events

Since its availability, the L4 mAb has been used to study the function of human P2X7, particularly the study of P2X7 channel activity and downstream events in vitro. As described above, this mAb can block human P2X7-mediated inward currents [10]. In line with this, the L4 mAb has been shown to block ATP-induced Ca^2+^ fluxes in HEK-hP2X7 cells [20, 21], and in human neural precursor cells and neuroblasts [22], ATP-induced Ba^2+^ fluxes in human chronic lymphocytic leukaemia (CLL) cells and ethidium^+^ uptake into normal human B cells [23], ATP-induced DAPI^2+^ uptake into HEK-hP2X7 cells [20] and ATP-induced YO-PRO-1^2+^ uptake into human myeloma RPMI 8226 cells [24]. In addition to human P2X7-mediated IL-1β release [10] and as mentioned in the introduction Di Virgilio and colleagues showed that the L4 mAb blocks multinucleated giant cell formation by human macrophages [11, 12]. Furthermore, they went on to show that this mAb can also impair osteoclast formation from human monocytes [25]. Di Virgilio and colleagues have also used this mAb to establish a role for P2X7-mediated inhibition of tolerogenic human leukocyte antigen (HLA)-G release from human monocytes [26]. Others have shown that the L4 mAb can impair ATP-induced alterations in membrane fluidity in human monocyte subsets [27], as well as ATP-induced phosphatidylserine exposure and CD62L shedding in human CD4^+^ T cells [20] and phospholipase D stimulation in human CLL cells [28]. Collectively, these studies highlight the utility of the L4 mAb to impair various P2X7-mediated events. Despite these observations, this mAb is used far less often to impair P2X7 activity compared to more widely available small molecule antagonists [29].

#### Immunoprecipitation

Based on the capacity of the L4 mAb to immunoprecipitate human P2X7 from XS63 cells [10], Ben Gu and the late James Wiley [30] and colleagues used L4 mAb-coated Dynabeads to immunoprecipitate human P2X7 from interferon (IFN)-γ/LPS-stimulated THP-1 cells and from HEK-hP2X7 cells [31]. These immunoprecipitate analyses revealed that cell-surface P2X7 is co-associated with myosin heavy chain IIa and myosin Va in these respective cell types. This and their subsequent studies established a novel role for P2X7 as a scavenger receptor, to mediate the phagocytosis of particles and dead cells in the absence of extracellular ATP [32].

#### Complement-dependent cytotoxicity

Based on the ability of murine IgG_2b_ to fix complement and lyse cells [33], the L4 mAb was shown to mediate the complement-dependent cytotoxicity of HEK-hP2X7 and RPMI 8226 cells, as well as various human blood leukocyte populations in vitro [21]. The relative amount of cytotoxicity was proportional to the relative amount of cell surface P2X7 in most leukocyte subsets. Under the conditions applied in this study, classical and intermediate monocytes, myeloid and plasmacytoid DCs, invariant natural killer T (iNKT) cells, myeloid-derived suppressor cell (MDSC) subsets, CD56^−^CD16^+^ natural killer (NK) cells and T helper (Th)17 cells were most sensitive to L4 mAb-mediated complement-dependent cytotoxicity. This study opens new areas of targeting P2X7^+^ cells with complement-fixing anti-P2X7 mAbs to selectively deplete these cells either in vitro*, *ex vivo or in vivo, to limit pathological P2X7-mediated responses.

#### In vivo administration

The L4 mAb has been used to block human P2X7 in vivo. Repeated intraperitoneal administration of this mAb reduced graft-versus-host disease (GVHD) progression in a humanised (xenogeneic) NOD-*scid* IL2Rg^null^ (NSG) mouse model, which was associated with increased human regulatory T cells (Tregs), natural killer T (NKT) cells and NK cells, and decreased Th17 cells [24]. As such, this study provided the first report of a biologic targeting human P2X7 in any context in vivo. Although the mechanism of action of this mAb in this study was not elucidated, based on work by Di Virgilio and colleagues that showed a role for P2X7 in mitochondrial stress during nutrient deprivation [34], the L4 mAb prevented the loss of human Tregs under nutrient deprivation (serum starvation) in vitro [24]. This suggests that the L4 mAb may have prevented the loss of human (donor) Tregs, which normally decline over time in NSG mice [35], to limit GVHD progression. It is unlikely that the effect of this mAb in vivo was due to complement-mediated depletion of human (donor) effector T cells, which mediate GVHD in NSG mice [36], as these mice lack complement activity due to a 2-base pair deletion in the *Hc* gene [37].

### Immunolabelling studies with the anti-human P2X7 mAb (clone L4)

#### Purification and conjugation

**Fig. 1:**
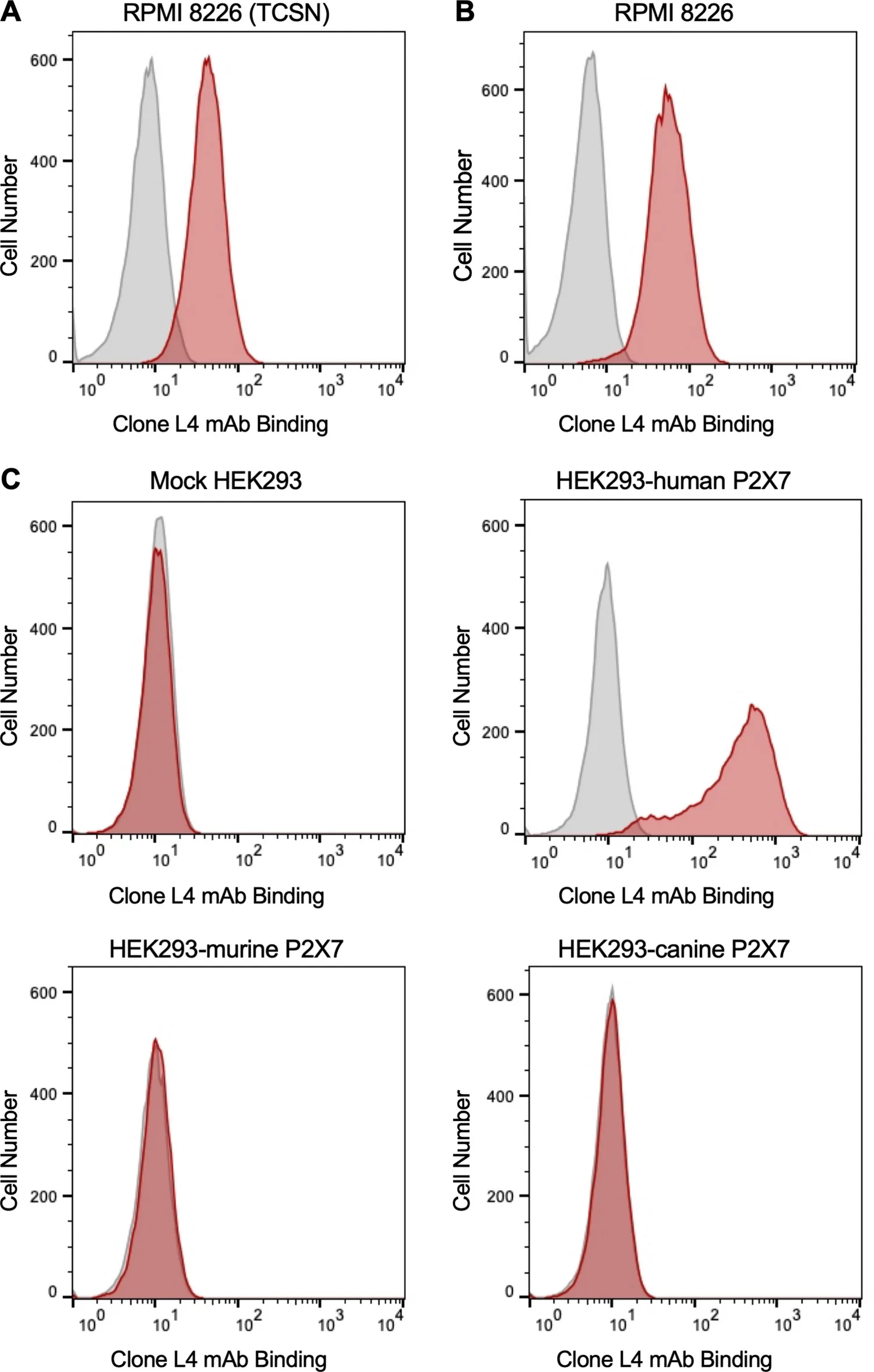
The anti-human P2X7 mAb (clone L4) binds human but not murine or canine P2X7. (**A**-**C**) The L4 mAb in tissue culture supernatant (TCSN) was purified via Protein A chromatography using a Millipore (Tullagreen, Ireland) Montage Antibody Purification Kit with PROSEP-A Media and concentrated to approximately 0.5 mg/mL using a Millipore Amicon Ultra 30 K device. (**A**,**B**) RPMI 8226 cells were prepared as described [38]. (**C**) HEK293 cells were transfected with plasmid DNA encoding human P2X7 [39], murine P2X7 [40] or canine P2X7 [41] as described [42]. (**A**-**C**) RPMI 8226 and HEK293 cells in PBS containing 10% foetal calf serum (PBS-FCS) were incubated with (**A**) L4 TCSN (red histogram) or (**B**,**C**) 0.5 μg purified L4 mAb (red histograms) or (**A**-**C**) 0.5 μg isotype control mAb (BD Biosciences) (grey histograms) for 20 min. Cells were washed once with PBS and incubated with 2 μL PE-conjugated sheep anti-murine IgG F(ab)’_2_ fraction (Chemicon, Melbourne, Australia) and 0.5 μg 7-aminoactinomycin D (Enzo Life Sciences, Plymouth Meeting, USA) in PBS-FCS for 20 min. Cells were washed once with PBS and analysed using a BD (San Diego, USA) LSR II flow cytometer as described [38]

The L4 mAb has been used mainly for immunolabelling, particularly for the detection of human P2X7 on cells by flow cytometry. The mAb is typically applied following IgG purification via Protein A chromatography [10, 23], but can be used as a hybridoma supernatant [10, 18] (Fig. 1A).

In relation to immunolabelling, the L4 mAb has been used with anti-murine secondary antibodies conjugated to fluorescein isothiocyanate (FITC) [10], Alexa Fluor™ 488 [43], phycoerythrin (PE) [44], Alexa Fluor™ 647 [20] and cyanine 5 (or Cy5) [45] for flow cytometry, cyanine 2 (or Cy2) for confocal microscopy [46], or biotin for immunohistochemistry [10]. In contrast, others have reported that the L4 mAb is not suitable for immunohistochemistry [47]. Thus, further testing of this mAb in this system is required. The purified mAb has also been directly conjugated to FITC [23], Alexa Fluor™ 488 [23], Alexa Fluor™ 647 [48] or DyLight™ 488 [17] for flow cytometry, or the metal conjugate ^141^Pr for mass cytometry [49].

Collectively, these studies demonstrate the versatility of the L4 mAb with several immunolabelling detection systems, which has led to the description of P2X7 in various cell types, as described in detail below. Before proceeding with this topic, two additional points should be noted. First, the L4 mAb was reported by Di Virgilio and colleagues to bind both the canonical form of human P2X7 (P2X7A), as well as the naturally occurring C-terminal truncated isoform P2X7B [50]. Thus, immunolabelling studies with the L4 mAb are unable to differentiate between either isoform. It remains unknown, however, if this mAb can block the P2X7B channel activity or events downstream of this isoform. Second, purified L4 mAb was studied as part of the Tenth Human Leucocyte Differentiation Antigen (HLDA) Workshop [51]. Key findings with the L4 mAb (code 10–70) from the workshop are included below. This mAb, as supplied to the workshop, was purified via Protein A chromatography and its binding to human P2X7 assessed using a PE-conjugated anti-murine secondary antibody and flow cytometry. The L4 mAb bound to RPMI-8226 (Fig. 1B) and HEK-hP2X7 cells but not mock-transfected HEK293 cells or HEK293 cells transfected with murine or canine P2X7 (Fig. 1C).

#### Peripheral blood leukocytes

The L4 mAb has been largely used to demonstrate the presence of cell-surface P2X7 on human peripheral blood mononuclear leukocyte subsets. It was initially shown using flow cytometry that P2X7 is present on these cells from healthy individuals with a rank order of expression (highest to lowest) on monocytes > B cells > NK cells > T cells [23] (Table 1). In this same study, a similar pattern was observed in these cell types from individuals with CLL. In contrast, minimal cell-surface P2X7 was present on neutrophils or platelets [23] but cell-surface P2X7 has been reported in both cell types in subsequent studies with the L4 mAb [54, 55]. Notably, the earlier study reported large pools of intracellular P2X7 in all examined cell types [23], the functional significance of which remains unknown. A subsequent study revealed that cell-surface P2X7 is present at similar amounts on human peripheral blood CD4^+^ and CD8^+^ T cells [56], while another confirmed greater amounts of cell-surface P2X7 on monocytes compared to NK cells [57].

**Table 1:** Rank order of cell-surface P2X7 on human leukocytes as revealed using the anti-human P2X7 mAb (clone L4) with flow cytometry

Rank order (highest to lowest) of cell-surface P2X7	Tissue	Fluorochrome	Reference
Monocytes > B cells > NK cells > T cells > neutrophils and platelets	Blood	FITC	[23]
Intermediate and classical monocytes > myeloid DCs, plasmacytoid DCs and iNKT cells > CD56^−^CD16^+^ NK cells and granulocytic MDSCs > non-classical monocytes, and CD56^+^CD16^+^ and CD56^hi^CD16^−^ NK cells, early and monocytic MDSCs > B cells, CD4^+^ and CD8^+^ T cells, and Tregs	Blood	DyLight™ 488	[21]
NKT, MAIT and γ δ T cells > conventional T cells	Blood and liver	Alexa Fluor^™^ 647	[52]
CMRF-56^+^ myeloid DCs > CD1c^+^ myeloid and plasmacytoid DCs > (absent) CD16^+^, CD34^+^ and CD141^+^ myeloid DCs	Blood	Alexa Fluor™ 488*	[43]
CD1c^+^ myeloid DCs > plasmacytoid and CD141^+^ myeloid DCs	Blood	PE*	[44]
CD11b^+^ conventional DCs > plasmacytoid DCs, HLA-DR^+^ non-DCs, HLA-DR^−^ thymocytes > CD141^+^ conventional DCs	Thymus	Cyanine 5*	[45]
Intermediate monocytes > SLAN^−^ non-classical monocytes > classical monocytes > SLAN^+^ non-classical monocytes	Blood	FITC*	[53]

^*^Detected using a fluorochrome-conjugated secondary antibody

Abbreviations: DCs, dendritic cells; FITC, fluorescein isothiocyanate; HLA, human leukocyte antigen; iNKT, invariant natural killer T; MAIT, mucosal-associated invariant; MDSCs, myeloid-derived suppressor cells; NK, natural killer; PE, phycoerythrin; SLAN, 6-sulfo N-acetylglucosamine; Tregs, regulatory T cells

**Fig. 2:**
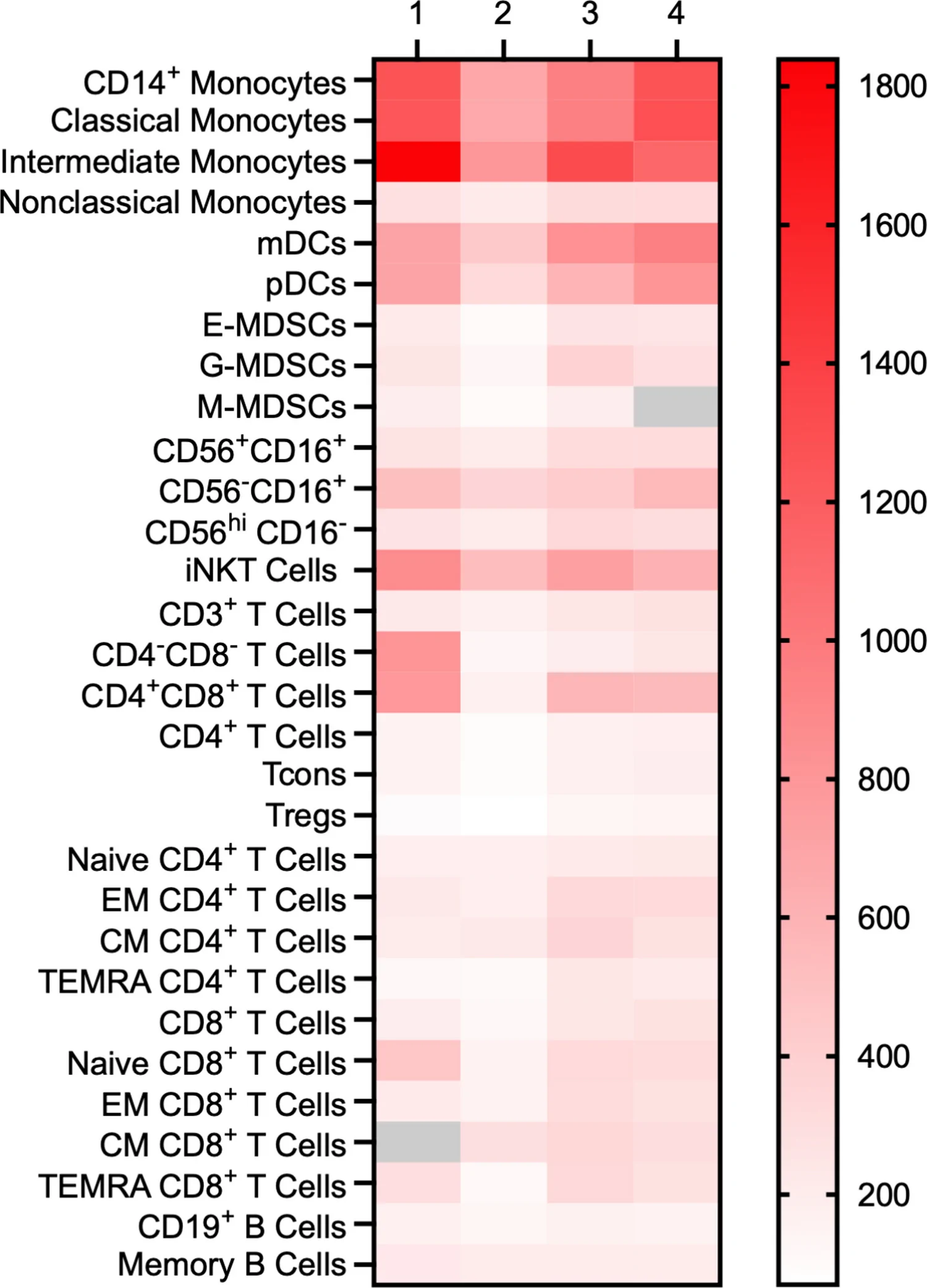
The anti-human P2X7 mAb (clone L4) reveals different amounts of cell-surface P2X7 on human mononuclear leukocytes. In a previous study [21], peripheral blood mononuclear leukocytes from four different donors (1–4) were isolated by density centrifugation, labelled with DyLight™ 488-conjugated L4 mAb and analysed by flow cytometry. This published data was replotted as a heatmap using Prism for macOS v11 (GraphPad Software, LLC. Boston, USA). Scale bar represents geometric mean fluorescence intensity of cell-surface P2X7. Grey cells represent missing data. Abbreviations: CM, central memory; E-MDSCs, early MDSCs; EM, effector memory; G-MDSCs, granulocytic MDSCs; iNKT, invariant NKT; M-MDSCs, monocytic MDSCs; mDCs, myeloid DCs; pDCs, plasmacytoid DCs; Tcons, conventional CD4^+^ T cells; TEMRA, terminally differentiated effector memory; Tregs, regulatory CD4^+^ T cells

In a recent flow cytometric study, use of the L4 mAb has revealed a more detailed analysis of cell-surface P2X7 on human blood mononuclear leukocytes from healthy individuals, with a rank order of expression (highest to lowest) on intermediate and classical monocytes > myeloid DCs, plasmacytoid DCs and iNKT cells > CD56^−^CD16^+^ NK cells and granulocytic MDSCs > non-classical monocytes, and CD56^+^CD16^+^ and CD56^hi^CD16^−^ NK cells, early and monocytic MDSCs > B cells, CD4^+^ and CD8^+^ T cells, and Tregs [21] (Fig. 2, Table 1). A similar pattern has been observed using the L4 mAb with mass cytometry, except for lower cell-surface P2X7 on plasmacytoid DCs and CD56^−^CD16^+^ NK cells, and more P2X7 on non-classical monocytes [58]. Both studies revealed similar amounts of P2X7 on naïve and memory CD4^+^ or CD8^+^ T cells, but greater amounts of P2X7 on memory B cells compared to naïve B cells [21, 58]. Consistent with the above findings in relation to conventional and innate T cells, the L4 mAb revealed greater amounts of cell-surface P2X7 on NKT cells, mucosal-associated invariant (or MAIT) cells and γ δ T cells compared to conventional T cells in the blood and livers from humans [52] (Table 1).

#### Dendritic cells

Di Virgilio and colleagues, using the L4 mAb with flow cytometry, were the first to demonstrate the presence of cell-surface P2X7 on human monocyte-derived DCs, with similar amounts of cell-surface P2X7 observed on LPS-primed monocyte-derived DCs [13]. Similar studies by others have confirmed the presence of cell-surface P2X7 on human monocyte-derived DCs [59, 60] and human monocyte-derived Langerhans cells (LCs) [61]. Notably, monocyte-derived LCs have lower P2X7 activity than that of monocyte-derived DCs generated from the same donors despite similar amounts of cell-surface P2X7 [61]. This reduced activity is associated with increased cell-surface CD39 on monocyte-derived LCs compared to monocyte-derived DCs [61], consistent with the notion that CD39 can negatively regulate P2X7 activation [62]. Finally, use of the L4 mAb has revealed that human monocyte-derived macrophages have a greater amount of cell-surface P2X7 than human monocyte-derived DCs [59, 60]. It remains to be tested if this reflects the pattern of P2X7 on macrophages and DCs within tissues.

Given these early observations, including that by Di Virgilio and colleagues [13], the presence of P2X7 on human DC populations was a major focus of the Tenth HLDA Workshop (Table 1). The most comprehensive study of blood DCs from this workshop revealed a rank order of cell-surface P2X7 (highest to lowest) on CMRF-56^+^ myeloid DCs > CD1c^+^ myeloid and plasmacytoid DCs > (absent) CD16^+^, CD34^+^ and CD141^+^ myeloid DCs [43]. A smaller study of blood DCs revealed a rank order of cell-surface P2X7 (highest to lowest) on CD1c^+^ myeloid DCs > plasmacytoid and CD141^+^ myeloid DCs [44], while another study confirmed the presence of P2X7 on myeloid but not plasmacytoid DCs [53]. In general, these findings support the notion that cell-surface P2X7 is greater on myeloid DCs than plasmacytoid DCs but also suggest cell-surface P2X7 varies between myeloid DC subsets. Finally, L4 mAb immunolabelling of human thymus samples revealed the presence of cell-surface P2X7 with a rank order (highest to lowest) on CD11b^+^ conventional DCs > plasmacytoid DCs, HLA-DR^+^ non-DCs, HLA-DR^−^ thymocytes > CD141^+^ conventional DCs [45]. This indicates that P2X7 is present in the human thymus and that the receptor is most likely subject to central T cell tolerance mechanisms.

#### Macrophages

An early study with the L4 mAb confirmed the presence of intracellular pools of P2X7 in monocytes and revealed that P2X7 is increased in monocyte-derived macrophages from day 4 of differentiation [63], a finding confirmed by others with this mAb [59]. Use of the L4 mAb, as part of the Tenth HLDA Workshop, revealed that cell-surface P2X7 is greater on granulocyte/macrophage-colony stimulating factor-differentiated human macrophages compared to macrophage-colony stimulating factor-differentiated human macrophages, with cell-surface P2X7 reported to be increased upon IFN-γ/LPS-stimulation in either cell type [60].

Consistent with the flow cytometric studies of human monocyte-derived macrophages and IL-1β release and immunoprecipitation studies of differentiated THP-1 cells outlined above, the L4 mAb can detect cell-surface P2X7 on IFN-γ/LPS-stimulated THP-1 cells but not undifferentiated THP-1 cells. Notably, this up-regulation can be prevented by co-incubation with transforming growth factor-β1 [64]. This suggests that cell-surface P2X7 in other cell lines could be manipulated by cytokines or other molecules. Collectively, this supports the concept that P2X7 is upregulated during differentiation and that its relative presence is influenced by the inflammatory milieu.

#### Monocytes

Monocyte subsets are typically reported as either CD14^hi^CD16^−^ classical, CD14^+^CD16^+^ intermediate or CD14^lo^CD16^+^ non-classical monocytes [65]. A report from the Tenth HLDA Workshop testing the L4 mAb revealed a rank order of cell-surface P2X7 (highest to lowest) on intermediate monocytes > 6-sulfo N-acetylactosamine (SLAN)^−^ non-classical monocytes > classical monocytes > SLAN^+^ non-classical monocytes [53] (Table 1). Since this time, SLAN^+^ cells are now considered to be mostly non-classical monocytes [66] while SLAN^−^ cells may be both non-classical and intermediate monocytes [67]. Given this, the workshop ranking largely aligns with other studies [21, 58].

The use of the L4 mAb to measure relative differences in cell-surface P2X7 on human blood leukocytes, especially monocytes, in disease is an emerging area of investigation. A comparison of liver transplant recipients who either rejected (immune non-tolerant) or tolerated (immune tolerant) the transplant following withdrawal of immunosuppression revealed increased amounts of cell-surface P2X7 on circulating CD14^+^CD16^+^ monocytes from immune non-tolerant but not immune tolerant recipients, despite similar amounts of cell-surface P2X7 on these cells from both groups during immunosuppression [68]. This is consistent with a role for P2X7 in the rejection of skin allografts in mice as shown in this same study.

Cell-surface P2X7 is also increased on monocytes, primarily CD14^+^CD16^−^ and CD14^+^CD16^+^ monocytes, from patients with sepsis but not on monocytes from surgery patients or healthy individuals [69]. Consistent with this, LPS stimulation increased P2X7 on monocytes. Notably, stimulation of monocytes with IFNγ, tumour necrosis factor or IL-6 did not increase P2X7, suggesting bacterial products rather than pro-inflammatory cytokines are primarily responsible for the up-regulation of monocyte P2X7 in sepsis [70]. Using a mouse model, this study also showed that increased P2X7 is associated with mitochondrial failure and inflammasome dysfunction, and increased mortality in sepsis.

Compared to healthy individuals, cell-surface P2X7 is increased on total, classical and CD14^+^CD4^+^ monocytes from age-related macular degeneration patients with choroidal neovascularisation but not in early-stage disease or other late-stage classifications [27]. This supports a role for monocyte P2X7 in age-related macular degeneration.

By comparison, in Alzheimer’s Disease (AD), cell-surface P2X7 is reduced on total monocytes, as well as on classical, intermediate and non-classical monocytes from β-amyloid^+^ individuals compared to β-amyloid^−^ individuals [54]. A similar pattern was seen in AD individuals with dementia compared to cognitive normal individuals, with amounts of cell-surface monocyte P2X7 in pre-clinical AD individuals like the former group. Somewhat similar patterns in cell-surface P2X7 were seen in neutrophils, NK cells and CD14^−^CD16^−^ lymphocytes (T and B cells). These changes were not associated with *P2RX7* gene single nucleotide polymorphisms (SNPs) suggesting that environmental factors may contribute to the down regulation of P2X7 during AD progression. Collectively, this highlights P2X7 as a potential therapeutic target or biomarker in this disease.

#### Other primary cell types

The L4 mAb with flow cytometry has been used to show the presence of cell-surface P2X7 on keratinocytes isolated from primary human skin samples [61]. Of note, cell-surface P2X7 was greater on keratinocytes than epidermal LCs from the same skin samples, while cell-surface CD39 was absent on keratinocytes but present on LCs. Combined this was associated with greater P2X7 pore activity on keratinocytes compared to LCs. The L4 mAb with flow cytometry has also been used to demonstrate the presence of cell-surface P2X7 on human neural precursor cells and neuroblasts [22]. Like peripheral blood leukocytes, these cells had large pools of intracellular P2X7. Finally, the use of equilibrium binding with the L4 mAb with flow cytometry has been used to demonstrate the presence of surface P2X7 on human platelets and platelet-derived extracellular vesicles [55]. This highlights the potential of using similar approaches to detect low amounts of P2X7 on cells considered P2X7^lo^.

#### Malignant cell lines

The L4 mAb with flow cytometry has revealed the presence of cell-surface P2X7 on various human malignant cells lines. These include myeloma RPMI 8226 cells [38, 47, 71], acute myelogenous leukaemia KG-1 cells [71], Hodgkin lymphoma KM-H2 cells [43] and melanoma SK-MEL-5 cells [47]. In support of the relevance of these findings in cell lines, the L4 mAb has also revealed cell-surface P2X7 on acute myeloid leukaemia cells from human samples [43]. In contrast, the same approaches found no or minimal cell-surface P2X7 on Burkitt’s lymphoma Raji and Ramos cells, acute monocytic leukaemia THP-1 cells, acute promyelocytic leukaemia HL-60 cells, erythroblast leukaemia HEL cells, histiocytic lymphoma U-937 cells, neuroblastoma Kelly cells, breast epithelial MCF-7 and MDA-MB-231 cells, colon epithelial Caco-2, COLO 201 and HCT-8 cells, prostate epithelial LNCaP, PC-3 cells or DU143 cells, and skin epithelial A431 and HaCaT cells [38, 43, 47]. One group also showed that the L4 mAb does not bind to non-functional P2X7 (nfP2X7) following its mobilisation to the surface of RPMI 8226 or SK-MEL-5 cells induced by high concentrations of extracellular ATP [47]. This is consistent with the notion that P2X7 and nfP2X7 have different structural conformations [72], highlighting the relevance of nfP2X7 as a therapeutic target in cancer immunotherapy including chimeric antigen receptor (or CAR)-T cells [73, 74].

### Genetic studies with the anti-human P2X7 mAb (clone L4)

The L4 mAb has aided the discovery and characterisation of several SNPs in the *P2RX7* gene, coding for loss of function (LOF) or gain of function (GOF) P2X7 variants. This mAb helped characterise the first identified LOF *P2RX7* SNP (rs3751143), which codes for a Glu^496^Ala variant [75]. Cell-surface P2X7 was comparable on T and B cells from control individuals (that is those homozygous for the non-mutant allele) and individuals homozygous for this SNP [75, 76]. However, subsequent studies with the L4 mAb indicated that cell-surface P2X7 is reduced on human monocytes [77], LPS-primed monocytes [78] and monocyte-derived macrophages [79] from homozygous individuals compared to corresponding cells from control individuals. Consistent with these observations, the L4 mAb revealed slightly lower cell-surface P2X7 on Ala^496^ P2X7 expressing HEK293 cells compared to HEK293 cells with control P2X7 [80]. Thus, reduced cell-surface P2X7 because of this common LOF SNP may contribute in part to decreased ATP-induced IL-1β and IL-18 release from LPS-primed monocytes [77, 78] and ATP-induced intracellular killing of mycobacterium in IFNγ-stimulated macrophages [79], and increased sensitivity to extrapulmonary tuberculosis [81].

Use of the L4 mAb also assisted the characterisation of another relatively common LOF *P2RX7* SNP (rs2230911), which codes for a Thr^357^Ser variant [48]. This mAb revealed similar cell-surface P2X7 on T and B cells from control individuals and homozygous individuals, as well as on HEK293 cells with control or mutant P2X7. This indicated that the LOF was a result of changes to receptor activity rather than cell-surface expression.

The L4 mAb helped characterise a rare LOF *P2RX7* SNP (rs1653624), which codes for a Ile^568^Asn variant, revealing markedly reduced cell-surface P2X7 but preserved amounts of intracellular P2X7 in HEK293 cells, consistent with impaired receptor trafficking to the plasma membrane [82]. This mAb also revealed decreased cell-surface P2X7 in T and B cells from heterozygous individuals compared to control individuals. In contrast to this variant, the L4 mAb is unable to bind to cell-surface or intracellular P2X7 containing the Arg^307^Gln variant, which arises from another rare LOF SNP (rs28360457), in HEK293 cells [83]. Furthermore, mAb binding to T and B cells from individuals heterozygous for this SNP is lower than that of cells from control individuals. Collectively, this indicated that the L4 mAb binds to an epitope involving the Arg^307^ residue. Consistent with these findings, a *P2RX7* haplotype, which promotes skipping of exons 7 and 8, to generate the P2X7L isoform, which excludes part of the extracellular domain, is unable to bind the L4 mAb [84].

The L4 mAb has also been used to study the impact of compound heterozygous SNPs in IFNγ-stimulated human macrophages [85]. In this study, macrophages from compound heterozygous individuals (Arg^307^Gln/Glu^496^Ala, Arg^307^Gln/Ile^568^Asn or Glu^496^Ala/Ile^568^Asn) had reduced cell-surface P2X7 compared to macrophages from control individuals, which corresponded to reduced ATP-induced pore activity and intracellular killing of mycobacterium. Further, cell-surface P2X7 and P2X7 activity on single heterozygous individuals was lower than that of control individuals but greater than that of compound heterozygous individuals, demonstrating a gene dosage effect of these SNPs.

The L4 mAb has aided the characterisation of different haplotypes of the *P2RX7* gene. This mAb revealed similar cell-surface P2X7 between monocytes from control individuals and individuals homozygous for one of two GOF haplotypes, termed P2X7-2 and P2X7-4 [39]. The GOF in P2X7-2 and P2X7-4 were conferred by a shared SNP (rs1718119) encoding an Ala^348^Thr variant. Two further SNPs (rs208294 and rs7958311) encoding His^155^Tyr and His^270^Arg variants, respectively, also contributed to the GOF in P2X7-4. In contrast, in a study focusing on the Ala^348^Thr mutation alone, the L4 mAb revealed an increased trend in cell-surface P2X7 on monocytes from individuals homozygous for this mutation compared to control individuals, as well as greater cell-surface P2X7 on Thr^348^ P2X7 expressing HEK293 cells compared to control cells [80]. Larger numbers of individuals with known *P2RX7* gene haplotypes are required to reconcile these differences.

Another study involving the L4 mAb explored a rare haplotype including two rare LOF variants [86]; the *P2RX7* SNP (rs28360447) coding a Gly^150^Arg variant [39, 87] and the *P2RX4* SNP (rs28360472) coding for a Tyr^315^Cys variant [88]. This study revealed reduced P2X7-mediated phagocytosis in Arg^150^ P2X7/Cys^315^ P2X4 expressing HEK293 cells compared to control P2X7/P2X4 expressing cells or Arg^150^ P2X7 expressing cells, but with similar L4 mAb binding to all three cell types [86]. This indicated such differences were due to altered P2X7 activity rather than cell-surface expression.

In addition to polymorphic variants and haplotypes, the L4 mAb has been used to study the impact of site-directed mutations and truncated variants of P2X7. As mentioned above, this mAb was employed to confirm the cell-surface expression of the naturally occurring C-terminal truncated isoform P2X7B [50]. Furthermore, the L4 mAb allowed the study of site-directed mutations (Lys^193^Ala and Lys^311^Ala) potentially involved in ATP binding and which result in reduced P2X7 activity but not cell-surface P2X7 [89]. Finally, the L4 mAb revealed that point mutations Cys^572^Gly, Arg^574^Gly and Phe^581^Gly in the C-terminus of rat P2X7, which reduced both BzATP-induced channel and pore activity, were a result of reduced receptor trafficking to the cell-surface [90]. As such, this study demonstrated the L4 mAb can bind rat P2X7, despite the original observation that it was unable to inhibit rat P2X7 activity [10]. This finding with the L4 mAb and rat P2X7 parallels another, no longer available, anti-human P2X7 mAb (clone B2) (Buell and Chessell, *unpublished*) which can bind human P2X7 but not impair its activity [75].

### Comparison of the anti-human P2X7 mAb (clone L4) to other P2X7 techniques

Comparisons of the L4 mAb to other P2X7 techniques are limited, with few comparisons within the same study available. Examples of such studies include comparisons of *P2RX7* mRNA to cell surface P2X7. Di Virgilio and colleagues originally demonstrated that relative *P2RX7* mRNA expression was similar in unprimed and LPS-primed monocyte-derived DCs, which reflected the similar amounts of surface P2X7 on these cells in distinct states of differentiation [13]. Others have shown that the near-absence and presence of *P2RX7* mRNA coincide with the absence and presence of cell-surface P2X7 in undifferentiated and differentiated THP-1 cells [64]. More recently, increased *P2RX7* mRNA expression in monocytes compared to NK cells was shown to match differences in surface P2X7 on these cells [57]. Collectively, these studies indicate that relative *P2RX7* mRNA expression and cell-surface P2X7 largely align.

Examples of other studies with the L4 mAb include comparisons of P2X7 activity to cell surface P2X7. Relative amounts of cell-surface P2X7 on monocytes, NK cells, T and B cells, neutrophils and platelets were shown to correlate with relative amounts of P2X7 activity (ATP-induced ethidium^+^ uptake) in these cell types [23]. Likewise, comparisons of two studies from the same laboratory, demonstrated that the greater amount of cell-surface P2X7 on iNKT cells compared to that of CD4^+^ and CD8^+^ T cells, and Tregs [21] parallels the relative P2X7 activity (ATP-induced YO-PRO-1^2+^ uptake) in these cell types [91]. These comparisons, however, are potentially influenced by the presence of *P2RX7* gene SNPs, which impact P2X7 activity and/or cell-surface expression as detailed above. Further, these comparisons may be influenced by ectonucleotidases, where increased cell-surface CD39 on monocyte-derived LCs is associated with reduced P2X7 activity (ATP-induced ethidium^+^ uptake) compared to monocyte-derived DCs generated from the same individuals despite similar amounts of surface P2X7 on these two DC subsets [61].

A final example includes comparisons of an anti-human P2X7 nanobody (Dano1) to the L4 mAb in relation to P2X7 blockade [20]. Compared to the mAb, the nanobody was 20- to 50-fold more potent in impairing ATP-induced Ca^2+^ fluxes and DAPI^2+^ uptake in HEK-hP2X7 cells and was superior in preventing ATP-induced phosphatidylserine exposure and CD62L shedding in human CD4^+^ T cells.

### Conclusions and Future Directions

**Fig. 3:**
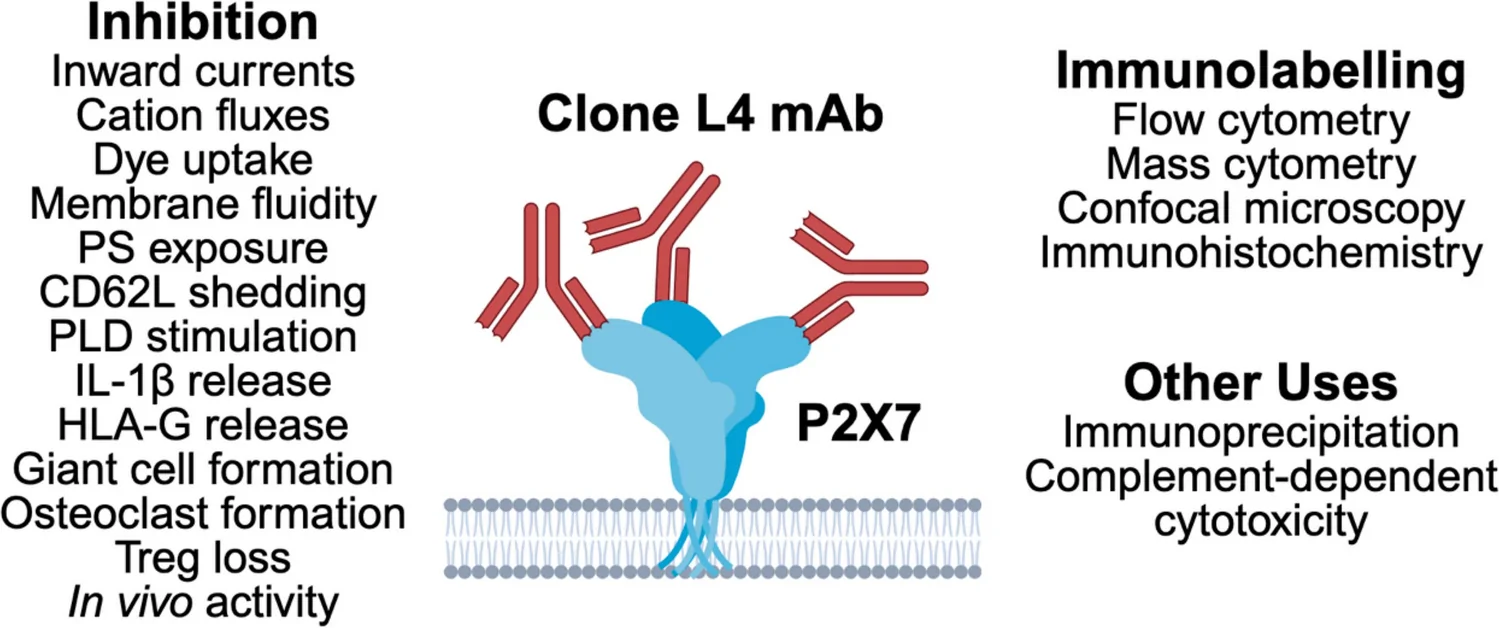
Reported applications of the anti-human P2X7 mAb (clone L4). The anti-human P2X7 mAb (or clone L4 mAb) can inhibit P2X7 activity and downstream events in vitro including inward currents, cation fluxes, membrane fluidity, phosphatidylserine (PS) exposure, CD62L shedding, phospholipase D (PLD) stimulation, interleukin (IL)−1β and HLA-G release, giant cell formation, osteoclast formation, and loss of Tregs under metabolic stress, as well P2X7 activity in vivo. The L4 mAb can also be used to immunolabel cells for analyses by flow and mass cytometry, confocal microscopy and immunohistochemistry. The L4 mAb can be applied to other techniques such as immunoprecipitation and complement-dependent cytotoxicity. Created with BioRender.com

Since its early adoption by Di Virgilio and colleagues, and others, the L4 mAb has helped define the distribution and function of human P2X7 in many cell types (Fig. 3). These pioneering and subsequent studies have revealed that the mAb can impair both P2X7 channel and pore activity, and various events downstream of P2X7 activation in vitro. However, the precise binding site of this mAb to human P2X7, including high-resolution cryo-electron microscopy images of the L4 mAb bound to human P2X7, remain to be resolved. Work in humanised mice has extended the use of the L4 mAb to in vivo studies revealing the potential of anti-P2X7 blocking mAbs or similar biologics as alternative therapeutic options to impair P2X7 in humans. Likewise, the use of this mAb in other humanised mouse models should be considered to explore further the role of human P2X7 in vivo. This approach could include the study of human P2X7^+^ tumour cells in xenograft models. The L4 mAb can be used to immunolabel P2X7 on cells for analyses by flow or mass cytometry, confocal microscopy, and immunohistochemistry, although the latter requires further development and confirmation. Future studies could explore how amounts of P2X7 detected using this mAb compare to other techniques especially gene expression techniques and to those techniques using other anti-P2X7 antibodies or nanobodies. Further to this, the potential exists to use the L4 mAb with quantitative flow cytometry or radioligand assays to quantify the number of P2X7 molecules of the surface of a given cell type. This mAb remains a useful tool to immunoprecipitate P2X7 for the identification of molecules co-associated with this receptor in different cell types and its use in other cell types should be considered to potentially find new human P2X7 binding partners, which may afford novel insights into P2X7 function. The L4 mAb provides new opportunities for the depletion of P2X7^+^ cells by complement-dependent cytotoxicity. Moreover, future studies should explore whether this mAb can also mediate phagocytosis or antigen-dependent mediated cytotoxicity of human P2X7^+^ cells. Finally, the L4 mAb should continue to assist with the characterisation of polymorphic and other variants of human P2X7. In this regard, an antibody or nanobody which can distinguish between P2X7A and P2X7B would advance the study of these isoforms. Researchers are encouraged to continue the use of the L4 mAb in future studies of P2X7.

## Data Availability

Data presented in the figures is available upon reasonable request.
